# Unfitness to plead in England and Wales: Historical development and contemporary dilemmas

**DOI:** 10.1177/0025802419856761

**Published:** 2019-06-15

**Authors:** Penelope Brown

**Affiliations:** 1 Institute of Psychiatry, Psychology and Neuroscience, King’s College London, UK; 2 South London and Maudsley NHS Foundation Trust, UK

**Keywords:** Expert witness, forensic psychiatry, human rights, fitness to plead, mentally disordered offenders, mental-health law

## Abstract

Fitness to plead refers to a criminal defendant’s ability to participate at trial. The purpose of fitness-to-plead laws is to protect the rights of vulnerable individuals who are unable to defend themselves in court and to preserve natural justice in the legal system while balancing the needs to see justice served and protection of the public. Early legal systems treated mentally disordered defendants with leniency, but over time those found unfit to plead have been subjected to indefinite incarceration, breaching their right to liberty while protecting their right to a fair trial. Conversely, the threshold for being found unfit is high, and there are concerns that many unfit defendants are being unfairly subjected to trial. The approaches to balancing the competing demands have changed over time and have led to confusing and contradictory practices. In order to understand better how and why the current problems have come to exist, this paper analyses the historical development of the legal framework for fitness to plead from Medieval England to the turn of the 21st century. It isolates core dilemmas: (a) what the normative standard of fitness to plead is and whether the current test for determining fitness adequately reflects this standard; (b) whether fitness to plead should be disability neutral or whether unfitness requires the presence of a psychiatric diagnosis; and (c) how the courts should deal with those found unfit to plead, including insuring against the deprivation of liberty of innocents while ensuring the public are adequately protected.

## Introduction

Fitness to plead is a fundamental but under-researched concept of criminal justice. It refers to a defendant’s ability to understand and participate in the legal processes within a criminal trial – a prerequisite of a fair trial. It has long been recognised that some defendants are not capable of so doing. Going ahead with trial would be an abuse not only of their individual rights but also of the rule of law.^1^ If a defendant cannot properly defend themselves, it could lead to inaccurate or unjust verdicts and, at worst, imprisonment for an offence which they did not commit.

The purpose of the law on fitness to plead is twofold: to balance the rights of vulnerable defendants with public protection and to balance natural justice with the need to see justice served. Any test for ‘unfitness’ should accurately and fairly distinguish those able to participate meaningfully in trial from those who cannot. At present, there are concerns in several jurisdictions that it fails to serve this purpose, with widespread consensus that many incapacitated defendants are slipping through the net and unfairly facing trial.^2–7^ Conversely, those who manage to meet the high threshold for unfitness are often unduly subjected to indefinite psychiatric detention, even when there is no evidence that they have a mental disorder.

In 2008, the Law Commission of England and Wales announced its intention to review fitness-to-plead law. In 2010, it published the first paper identifying problems in the existing framework, including that the test seems to overlook defendants’ abilities to make autonomous decisions and is less stringent than the civil test for decision-making capacity in the Mental Capacity Act 2005 (MCA).^8^ Following an iterative consultation, a final report and draft legislation published in 2016 outlined a new capacity-based test which potentially lowers the threshold for unfitness.^9,10^ The recommendations have been broadly welcomed,^11^ but are also challenged by a concurrent development in human rights law.^12^ The United Nations Convention on the Rights of Persons with Disabilities (UNCRPD) came into force in 2008 and requires member states to ‘recognize that persons with disabilities enjoy legal capacity on an equal basis with others in all aspects of life’.^13^ Findings of unfitness are primarily only available to those lacking mental abilities, resulting, for this group of defendants, in the denial of legal capacity on an equal basis with others. There are suggestions that fitness-to-plead laws and declarations of unfitness should accordingly be abandoned altogether.^14–16^

The concept of fitness to plead dates back centuries, and early English law forms the basis of procedures in many common-law jurisdictions. The following narrative explores how the judiciary in England and Wales has balanced the competing demands of justice, public protection and individual rights in assessing fitness to plead and dealing with those found unfit, starting from the 12th-century King’s Courts, through 19th- and 20th-century case law and statute, up to the turn of the 21st century. While this paper does not critique the recent proposed changes, including the influence of the MCA and UNCRPD, it aims to isolate dilemmas which emerged over time and led to concerns that the law is unfit for purpose, namely: how ‘fit’ one need to be to be fit to plead; whether mental disorder is necessary for unfitness; and how those found unfit to face trial should be dealt with.

## Early origins of fitness to plead

The concepts of crime and punishment have existed since civilised human life has been recorded, and offenders with mental disorders have long been granted special treatment in law. In the 4th century BC, Aristotle considered the circumstances in which a person may not be deemed culpable, which included acting due to a mistaken belief caused by ‘madness’.^17^ Ancient Roman law viewed suffering from mental illness as punishment enough for criminal behaviour: *satis furore ipso puniter.*^18^ In pre-Norman England, a perpetrator unable to understand the nature of a crime was deemed unable to form the necessary intention required for guilt (*mens rea*), even if they had committed the criminal act (*actus reus*). Such ‘insane’ defendants were released to the care of their families rather than punished.^19^

Trial by jury was introduced in England after the Norman Conquest, and by the 13th century, the King’s courts were established. Defendants were confronted by their accuser before a jury decided whether they should be held to account.^20^ A conviction led to punishment, including forfeiture of property to the Crown. The accused was required to say ‘guilty’ or ‘not guilty’ in reply to the indictment. If they could not answer, then they could not be held answerable. The legitimacy of the trial was called into question,^21^ and the courts had to deal with those who could not, or would not, enter a plea. Such defendants were said to ‘stand mute’, and a jury was called on to establish whether they were ‘mute of malice’ or ‘mute by the visitation of God’.^22^ The former referred to malingering – wilfully withholding a plea if it appeared advantageous. Malingerers were subjected to *peine forte et dure* – starved and pressed under heavy stones until they capitulated (or, in some cases, were crushed to death).^23^ Those found mute by visitation were deemed to unable to plead, and were excused from trial and punishment. Mute by visitation was invariably associated with mental disorder, about which understanding was then embryonic and invariably thought to be caused by demonic or holy influences.^24^ This was reflected in the archaic terms used: ‘idiots’ referred to those with a cognitive disorder from birth; ‘the insane’ was a broad description of those who developed madness later in life, with ‘lunatics’ sometimes used to refer to those who alternated between madness and lucidity; and ‘deaf mutes’ had speech and hearing impediments, often without mental illness.^24^ All were conflated with the term ‘insanity’, and all could lead to mute by visitation, hence unfitness to plead.

## Eighteenth- and nineteenth-century case law

From the early 18th century, an adversarial criminal process evolved, and defendants took a more active role at court. This, alongside the writings of Sir Matthew Hale, shaped the development of fitness-to-plead procedures.^25^ Hale, a 17th-century legal scholar with a progressive grasp of mental disorder, was interested in the causal nexus, the behaviour caused by the disorder, and rejected a status-based approach whereby the mere presence of insanity would be enough to render one unfit. He proposed a functional model focusing on what defendants could do rather than what they could not. He distinguished the ‘absolute mad’, whom he viewed as exempt from criminal responsibility, from the partially insane who were not.^26^ He disaggregated deaf-mutism from insanity and submitted that deaf mutes should not be found unfit unless they were also mentally defective. Hale also viewed unfitness as temporary rather than a final outcome, and suggested trials be postponed until the insanity abated.^23,27^ Despite his influence, Hale’s approach was not initially embraced by the courts.^20^

From the mid-18th century, Hale’s writings gained prominence as fitness to plead became considered more than just the ability to enter a plea.^28^ In 1756, Dyle was charged with murder but appeared incapable of ‘attending to the evidence’.^20^ His lawyer could not take instruction from him. The jury deemed him ‘not of sound mind and memory’ and his trial did not proceed. In 1790, Frith was charged with high treason by throwing a stone at a coach conveying King George III. In considering fitness to plead, the Lord Chief Justice declared ‘no man shall be called upon to make his defence at a time when his mind is in that situation as not to appear capable of so doing’.^29^ In 1830, Esther Dyson, a deaf mute, was charged with murdering her child. Informed by Hale’s treatise, the judge instructed the jury to consider ‘if they were satisfied that the prisoner had not then, from the defect of her faculties, intelligence enough to understand the nature of the proceedings against her’.^30^ As Dyson could not challenge the jury or understand proceedings, she was found ‘insane’, spared trial and detained indefinitely.

## The case of Pritchard 1836

Pritchard was a deaf mute indicted for bestiality, a capital offence.^26^ Due to communication deficits, he did not enter a plea. He was found mute by visitation but, when subsequently asked to answer to the indictment, used a sign to indicate ‘not guilty’. The jury decided he was now able to plead, but the judge, Baron Alderson, suggested that simply being *able* to plead did not equate to being *fit* to plead. Proposing both a status-based and functional test, he asked the jury to first find whether Pritchard was ‘sane or not’ and then consider three elements:

First, whether the prisoner is mute of malice or not; secondly, whether he can plead to the indictment or not; thirdly, whether he is of sufficient intellect to comprehend the course of proceedings on the trial, so as to make a proper defence – to know that he might challenge any of you to whom he may object – and to comprehend the details of the evidence.^31^

Pritchard’s ability to instruct counsel was not considered because access to legal advice was not routinely available until later that century.^22^ This criterion arose in *Davies* (1853), found unfit as he could not properly instruct counsel due to mental illness,^32^ and was incorporated into the Pritchard test.

The Pritchard criteria were rapidly and repeatedly adopted as the legal standard for fitness to plead, but issues have arisen which challenge whether they justly assess the abilities needed to participate in a trial. In Pritchard, Alderson made insanity a necessary condition for unfitness before summarising the functional abilities required for trial. But do these criteria reflect the normative conditions necessary and sufficient for a fair trial? Second, is it necessary to be insane to fail to meet these conditions? Despite evidence suggesting he was neither ‘insane’ nor unable to plead, Pritchard was deemed unfit and then indefinitely detained. How, therefore, does the law serve its purpose of protecting natural justice and individual rights?

## Issue 1: the ‘fitness’ threshold

The philosopher Anthony Duff describes the normative dimensions of trial as much more than simply understanding the facts and entering a plea.^21^ A defendant is called to answer not only to the charge but to the court, the Crown, and to fellow citizens. The accusation is one of criminal conduct, unlawful not just immoral; a conviction is not merely a finding of fact but condemnation in the form of punishment. While basic cognitive and intellectual capacities are required for the factual dimensions, Duff proclaims these alone are not sufficient for fitness to plead. To engage at trial properly, the defendant must also understand the reasons not to have done the deed, the moral, emotional and criminal aspects of the act, and the prudential reasons for avoiding punishment. An accused who cannot comprehend the facts or communicate their wishes is clearly not fit to plead. One who comprehends the facts but is not ‘rational’ and is unable to grasp the normative dimensions should also be found unfit.^1^

Duff’s threshold for ‘fitness’ is high, and the extent to which most individuals fulfil these dimensions, regardless of mental disorder, is questionable. Many defendants have only rudimentary understanding of the adversarial process, and most require significant assistance from lawyers.^27^ Duff does not consider the abilities required to engage or refuse counsel, but these are fundamental.

In his review of early cases of fitness to plead, Grubin notes that the precedent set by *Dyson* and *Pritchard* resulted in a test of cognitive and communicative abilities, without considering rational thinking and decision-making capacity.^20^ As we have seen, the early formulations of the Pritchard test included an ability to instruct counsel properly and to make a proper defence. But how should ‘proper’ be interpreted according to the normative standard for fitness? Subsequent judgments have tended to ignore this issue,^33–35^ as discussed below, focusing on the cognitive dimensions and effectively lowering the standard for fitness.

Peay has proposed an alternative list of ‘core competencies’ underpinning the normative dimensions of trial, fleshing out the Pritchard criteria to consider the subtleties within each ability.^27^ The ability to instruct counsel includes organisational skills, autonomy in decision making and acknowledgement of impartiality in the justice system; the ability to give evidence comprises not only communication and language skills (with or without the assistance), but also appreciation of truth, false beliefs and lies, and an ability to speak without displaying symptoms which could prejudice a jury’s assessment of guilt. These competencies are arguably easier to discern than Duff’s moral norms, but they assume mental disorder is present in those found unfit, for which special expertise would be needed. But is there, or should there be, a diagnostic threshold or causative nexus for unfitness as there is for incapacity in civil settings according to the MCA?

## Issue 2: the diagnostic threshold

The test for fitness to plead is often regarded as ‘disability neutral’.^10^ Yet, insanity has been enmeshed with unfitness from the outset. It is arguable that for practical reasons, a diagnostic requirement for unfitness is needed. Countless defendants would be deemed unfit if Duff’s normative requirements for a fair trial were applied literally. This dilemma was considered by Hamblin Smith, a late-Victorian prison doctor who questioned:

… how many prisoners are capable of making what may reasonably be called a ‘proper defence’, or … of giving proper instructions for their defence. But it is clear that mere ignorance, or lack of education, or ordinary stupidity, will not be enough to justify a verdict of unfitness to plead.^36^

A diagnosis of ‘insanity’ was certainly an implicit requirement for unfitness in early cases, but insanity has been understood to mean different things over time. Grubin blames early unfitness judgments for perpetuating the broad definition of insanity which encompassed other disorders such as low intellect and communication deficits. The courts gave little consideration to how insanity impacted on the Pritchard criteria. The timing of the disorder was, however, significant. ‘Insanity on arraignment’ was unfitness to plead, and insanity at the time of the offence made the defendant eligible for a ‘not guilty by reason of insanity’ verdict.^26^ We now clearly differentiate the concepts of fitness to plead and criminal responsibility, but initially the distinction was purely temporal. Insanity was crudely defined by social norms and lay perceptions of madness.^26^ The problem of coupling it with unfitness with would barely have been evident when treatment was not an option. It suited the early Victorian era, where the legalistic attitude of detaining the mentally ill prevailed and cases could be dealt with rapidly without the need for a public trial.^37,38^ However, as society’s response to madness shifted to medicalism and treatment became a possibility, problems arose.

If unfitness to plead requires a diagnosis, who is best placed to make it? Lawyers and doctors had differing opinions. The former were suspicious of the latter, with one judge quoted as saying (in 1888): ‘When trial by medical men comes into vogue, well and good; but so long as trial by jury is the law of the land, I will not allow a medical man to be substituted for the jury’.^39^ Doctors, on the other hand, asserted that insanity should ‘never be decided without medical evidence’.^40^ There were no medical experts in *Pritchard,* although they were used in late 19th-century cases. When expert witnesses were called, the ‘ultimate issue’ rule applied. Medics could only report facts upon which opinions relating to symptoms and diagnosis were formed. They could not give an opinion on fitness to plead, including the defendant’s abilities according to the Pritchard criteria. Medical evidence was rarely contested, suggesting a united medical and legal approach to unfitness, but since medical evidence became mandatory in 1991, marked differences in opinion between psychiatry and the law have surfaced.

Pritchard was found unfit due to low intellect and deaf-mutism, not mental disorder. These are not conditions that require expert psychiatric evidence. A core purpose of fitness-to-plead law is to protect any defendant who cannot fairly participate in a trial, not only those with a diagnosable disorder. Yet, psychiatry is now heavily intertwined with fitness to plead, not only in determining the issue but also in dealing with the outcome.

## Issue 3: the detention threshold

If unfitness is due to a medical disorder, then surely a medical solution is required. Yet, for many years, the only outcome was incarceration. In 1800, James Hadfield attempted to assassinate King George III and was acquitted on the grounds of insanity.^41^ To protect the public from the criminally insane, the Criminal Lunatics Act 1800 (CLA) was passed which allowed the detention of those found unfit to plead and those not guilty by reason of insanity. Detention was predominantly in prisons or Victorian asylums which consisted primarily of restraint and sedation akin to punishment.^37^ It is hard to see how being found unfit was a protection of individual rights. The link between insanity and unfitness was strong. Yet, there was no consideration of a causal nexus, no investigation as to whether detention was necessary and no contemplation of treatment.

## Fitness to plead in the 20th century

The 20th century was a time of considerable change in psychiatry. Advances in the understanding and treatment of mental disorder were heralded, and the archaic concepts of ‘madness’ and ‘insanity’ disaggregated into diagnostically distinct conditions. Sensory impairments such as deaf-mutism were no longer considered mental disorders, and intellectual impairments were separated from mental illness. The Mental Health Act 1959 (MHA) granted increased powers for both controlling and treating the mentally ill.^42^ Procedural issues relating to fitness to plead were clarified in case law and statutory changes.

The issue of when unfitness should be raised was first considered in *R* v *Roberts* [1953], a deaf mute charged with murder.^43^ The defence, believing there were grounds to support a ‘not guilty’ plea, were reluctant to raise unfitness in the hope of an acquittal. The prosecution insisted unfitness be tried first: if found unfit, Roberts would be indefinitely detained. The judge held that the general issue should be heard first. Otherwise, it might result in ‘the grave injustice of detaining as a criminal lunatic a man who was innocent’. This was not followed in *Beynon*,^44^ where it was held that fitness to plead should be tried before the general issue, even if it were not raised by the prosecution or defence and even if it resulted in incarceration. The judge described it as the court’s ‘duty’ to try the issue of fitness to plead if the defendant appeared insane, even though this paradoxically resulted in violation of the defendant’s rights.

In 1963, the Criminal Law Committee reviewed and made recommendations to codify fitness-to-plead procedures,^45^ many of which were swiftly implemented in the Criminal Procedure (Insanity) Act 1964 (CP(I)A). The CP(I)A clarified that unfitness could be raised by the prosecution, who had to prove beyond reasonable doubt that the defendant is fit;^35^ the defence, who bear the burden to prove unfitness on the balance of probabilities;^46^ and the court, when the burden would be on the prosecution to disprove unfitness.^47^ The issue had to be raised as soon as it arose but could be postponed until the opening of the case for the defence so that the jury could return a verdict of acquittal if there was insufficient evidence of guilt. It could be raised even if the defendant entered a plea, and it was up to the jury to determine unfitness.^48^

Confusingly, the CP(I)A introduced the term ‘under disability in relation to a trial’ to describe unfitness. This not only strengthened the status-based approach to assessing fitness to plead, but also contributed to disposals being targeted at those for whom a disability was present. The only disposal that was considered was psychiatric. Under the MHA, unfit defendants could now be detained against their will in asylums or ‘special hospitals’ under a hospital order which provided treatment as well as public protection.^49^ But psychiatric care was not always necessary or appropriate, especially in cases of unfitness due to deaf-mutism. The committee recommended the courts have discretion when imposing hospital orders, but this was not written into statute. They also considered introducing a ‘Trial of Facts’ whereby the guilt of the accused could be probed using the available evidence, despite the defendant being unfit to plead. This would avoid the unacceptable scenario of indefinite detention of an innocent person but was rejected on the basis that it would be unacceptable to try a person who has been found unfit. There was already opportunity for acquittal if, following the prosecution’s evidence, there was no case to answer. The only option of dealing with an unfit defendant, other than acquittal, remained indefinite hospital detention. This disincentivised the defence to raise unfitness, other than in murder trials where the alternative if found guilty was the death penalty, and a steady decline in the numbers found unfit ensued.^50^

Rather than balancing the core dilemmas of fitness to plead, the CP(I)A represented a ‘blurred compromise’ between medicalism and legalism.^42^ While it sought to prevent the detention of innocents, it failed to go far enough to protect individual rights. The only realistic outcome of being found unfit – indefinite hospitalisation – was decided by the courts rather than clinicians, and was invariably not in the interests of the accused. Admission had to occur within two months of the order being made, and in the meantime, the defendant could be held in a place of safety, primarily prison. Once in hospital, the defendant fell under the framework of the MHA 1959, and there was no provision to appeal. It did not provide any changes to the Pritchard criteria, for which it was heavily criticised.^51^

In 1972, the Butler Committee carried out a further review of the laws pertaining to mentally disordered defendants. The guiding principle of the resultant Butler Report was ‘treatment wherever possible’.^52^ To address the problem of detaining an innocent but unfit defendant, they recommended flexible disposal options, as well as delaying the trial to enable the individual to be restored to health. They developed the idea of a ‘Trial of Facts’, and suggested that those found to have ‘done the act’ charged against them should be considered ‘guilty in all but name’ (as it would not be right to find someone guilty when they have not had a full trial). Those found not to have ‘done the act’ should be discharged. They did not suggest a root and branch reform of the law, and were criticised for failing to recommend any changes to the Pritchard criteria.^42,53^ The report received a mixed reception, and many of the recommendations were deemed too controversial for immediate implementation.^53,54^

## Reform in the late 20th century

In the 1970s and 1980s, many defendants were still found unfit for reasons unrelated to mental illness, including deafness and communication difficulties.^55^ Psychiatric detention was not only inappropriate, it became unlawful. Under Article 5 of the European Convention on Human Rights (ECHR), deprivation of liberty due to psychiatric detention is only permitted if supported by objective medical expertise that there is a mental disorder warranting confinement.^56^ Yet, there was no provision for the court to distinguish between a finding of unfitness and an appropriate disposal. In *X* v *UK*, it was found that detention under the MHA 1959 contravened the right to liberty, as there was no access to review by a judicial body.^57^ This triggered a complete overhaul of mental-health legislation, which resulted in the MHA 1983.^58^ This was heralded as a major reform in the treatment of the mentally ill,^59^ emphasising patients’ rights, including the right to appeal against detention. A tribunal now had the power to discharge patients, even those detained by the courts, providing it was satisfied that the criteria for detention were no longer met. While this was an improvement for unfit defendants, the rights of the individual were often overshadowed by the need to protect the public when appeals were heard.^60^ Furthermore, the only safeguard available to unfit defendants remained hospitalisation, which defied the principle of protecting individual rights within fitness-to-plead procedures. Two cases in the 1980s highlighted this paradox and prompted further reform.^61^

In 1985, Glen Pearson, a deaf mute with significant learning disability, was charged with stealing five pounds and three light bulbs.^62^ Pearson was found unfit and to have done the act. Despite psychiatric reports stating that he was not insane, the only option was hospital order. A bed could not be immediately identified. So, a ‘place of safety’ was found in Lincoln Prison. Pearson was detained for three months, and this caused a national outcry. In Parliament, the procedure for dealing with unfitness was described as being ‘as remorseless in its purpose as anything out of a Greek tragedy’.^63^

The case and additional issues have been summarised by Emmins.^63^ First, the court had no option but to make a hospital order, despite it being evident that Pearson did not fulfil the criteria for detention. Even if he had, what was to be gained? His lack of intelligence and deafness were not likely to respond to treatment, and he did not pose a risk to others. Had he been tried and found guilty, he probably would have been given a fine. Second, it was apparent that there was no option for unfit defendants to be remitted for trial if their mental condition improves. This emerged in 1989, when a policy to review fitness every six months for the first two years of detention was introduced.^64^ Third, when hospitalisation is not appropriate, there is no other provision for protecting the public from unfit defendants. Three months after Pearson was given a restricted hospital order, he was discharged and went to live with his parents. This would have been most concerning if the charge against him was one which put the public at a risk. The tribunal can and does consider public safety when deciding whether to discharge a restricted patient, but it remains under a duty to discharge if the medical criteria for detention are not met. Its decision cannot be overridden, and the individual could not then be brought back to trial. How could the public be protected in cases of unfitness when hospital is inappropriate but there is significant risk to others? In view of the fact that one purpose of considering fitness to plead is to balance individual rights with public protection, the legal framework in England and Wales at the end of the 20th century was failing to meet either of these competing demands.

How did this situation come about? Medicine had progressed significantly, and there was now a nuanced understanding of what had been previously been clumped together as ‘insanity’, but this was not reflected in the law. The MHA 1983 differentiated between mental illness, mental impairment (or learning disability) and psychopathic disorder, and considered whether disorders were treatable when deciding to admit a patient to hospital. Yet, the broad concept of insanity from the time of Pritchard, now referred to as ‘under disability’, persisted in the fitness-to-plead framework. Psychiatric hospitalisation of an unfit defendant makes sense if that individual suffers from a mental disorder, but unfitness per se is not a psychiatric condition.^20^ It does not require the presence of mental disorder, and the Pritchard test detects unfitness in those in whom there is none.^50^ Yet, if unfitness is something for which only psychiatrists are qualified to assess and only psychiatric disposals are available, then the test for unfitness should better align with psychiatric thinking. However, a purely psychiatric model would overlook other reasons why a defendant cannot participate meaningfully in their trial, such as intellectual immaturity. It would not accurately and fairly distinguish those fit to plead from all those who are not.

Returning to the 1980s, the dilemma of dealing with an unfit but innocent defendant remained an issue which the government was forced to address following the case of Valerie Hodgson.^65^ Hodgson had severe learning disabilities and lived with her father. One day, she found him stabbed in the chest and confessed to his murder. She was found unfit and detained in a secure hospital for 14 months. It later transpired that her nephew had carried out the stabbing.^66^ In response to further outcry, the Home Office commissioned Prof Ronnie Mackay to review all cases of unfitness in the preceding decades. Mackay found a significant decline in findings of unfitness in the years following the CP(I)A.^50^ In 1989, only 11 cases were identified nationally. Mackay also found that while many of the findings of unfitness related to serious charges such as murder and rape, almost a quarter were in minor cases. The absence of establishing guilt and the prospect of indefinite detention were likely explanations for the reluctance to raise the issue.

In 1991, the Criminal Procedure (Insanity and Unfitness to Plead) Act was passed and amended the CP(I)A. After previous stalled attempts, the ‘Trial of Facts’ was finally introduced, allowing a jury to determine whether they are satisfied that the accused ‘did the act or made the omission charged against him’. Unlike a criminal trial, this hearing considers only *actus reus*, not *mens rea.* If *actus reus* cannot be proven, then the defendant is acquitted. For those found to have done the act, the amendments now allowed the court to order guardianship, supervision and treatment, or even absolute discharge, as alternatives to a hospital order. However, when the charge was murder, an offence for which the sentence was fixed by law, a hospital order with restrictions remained the only available disposal following a finding of unfitness.

The flexible disposal options were much more satisfactory, and the 1990s saw a marked upturn in findings of unfitness from 13 cases in 1990 to 80 in 2000.^55,67^ The CP(I)A amendments also introduced the requirement for medical evidence from two practitioners (usually psychiatrists) to support a finding of unfitness. Fitness to plead was increasingly becoming a psychiatric issue in the eyes of the law, but as psychiatry advanced into the 21st century, the test was stuck in 18th-century precedent which did not make a good fit.

## Interpreting Pritchard

The Pritchard criteria continue to provide the accepted test for fitness to plead to this day. The test is not codified in statute but was expounded in the 2003 case of *M (John)* as assessing the defendant’s abilities to understand the charges, decide whether to plead guilty, exercise the right to challenge a juror, instruct solicitors and counsel, follow the course of proceedings and give evidence in his/her own defence.^68^

Despite no explicit diagnostic criterion, there remains an implicit requirement that some form of disability is present for a finding of unfitness. In the first unfitness case heard at the Court of Appeal, Emery, an uneducated deaf mute, was deemed unable to understand and follow proceedings, resulting in a finding of unfitness and indefinite incarceration.^69^ His lawyer appealed that there was no evidence of insanity in terms of mental disorder. Therefore, the CLA should not apply to him, and he should have been found fit. The appeal was rejected. Lord Alderstone held ‘there was no question of general insanity, but only of insanity from the point of view of not understanding the nature of the proceedings’. The broad diagnostic threshold persists, as almost a century later, the judge in *R* v *M (John)* directed the jury not only to consider whether the defendant met the criteria listed above, but also to be satisfied that he was ‘suffering from a disability which rendered him unfit’.^68^ The Law Commission recently claimed that the Pritchard test includes ‘no diagnostic requirement’,^10^ but case law suggests there is not only a requirement for a diagnosis for unfitness but also a causative nexus.

Whether disability is necessary for a finding of unfitness is debatable; it is certainly not sufficient. The threshold for unfitness depends on how the functional criteria are read, in particular the interpretation of ‘proper’. A frequently cited example is the case of *Robertson*,^35^ charged with murder having experienced persecutory delusions that he was being poisoned. He had a clear understanding of legal procedure, but his delusions were thought to affect his ability to conduct his defence properly and led him to act ‘outside his own best interests’. He was initially found unfit, but on appeal, this finding was quashed: acting in one’s own interests is not part of the criteria. But what about the need to make a proper defence? Lord Parker reasoned that Robertson had:

… a complete understanding of the legal proceedings and all that is involved and, although he suffers from delusions which at any moment might interfere with a proper action on his part, that is not a matter which should deprive him of his right of being tried.^35^

A similar conclusion was reached in *Berry* who had paranoid schizophrenia and was described as being in a ‘grossly abnormal mental state’.^34^ A finding of unfitness was overturned on appeal, as Berry was deemed able to challenge jurors, instruct counsel, understand the evidence and give evidence, without consideration of whether he was acting rationally or in his best interests. Lord Lane noted that ‘a high degree of abnormality does not mean that the man is incapable of following a trial or giving evidence or instructing counsel and so on’. That such significant paranoia and delusions do not lead to legal findings of unfitness is at odds with how we now understand mental illness and mental capacity. Psychosis does not necessarily affect basic cognitive functions such as understanding evidence or entering a plea in the same way that learning disability can, but psychotic symptoms, especially delusions, have been found to impact decision-making abilities significantly in other settings.^70^ Hale understood this in the 17th century. Baron Alderson’s formulation required consideration of whether insanity affected the ability to defend oneself properly. Yet, throughout the following centuries, the courts relied on the more cognitive abilities in the Pritchard test (intellect and understanding), without considering how properly or rationally the defendant can make decisions and act. Indeed, the current widely accepted version of the Pritchard test as laid out in *M (John)* has dropped the term ‘proper’ altogether.^68^

The problem is compounded, as deficits in ‘cognitive’ abilities are less treatable than symptoms of mental illness such as delusions, paranoia and hallucinations. Hospital admission is appropriate if the purpose is for treatment, rather than purely public protection. Yet, the test for fitness to plead does not consider this. It is counter-intuitive that psychiatrists are called upon to give evidence, and psychiatric hospitals are the mainstay for managing individuals whose difficulties are with defending themselves at court rather than mental disorder as defined by the MHA. At the time of Pritchard, the concepts of autonomy and best interests would not have held significance, but they do now. However, the courts have repeatedly employed a narrow, cognitive interpretation of the criteria, rendering the threshold for unfitness high, arguably too high for the 21st century.^3^

## Human rights and fitness to plead

The turn of the 21st century saw the ECHR partially incorporated into English law for the first time with the passing of the Human Rights Act 1998. The need to safeguard fundamental rights gained prominence, and the fitness-to-plead framework came under scrutiny.^71^ Protecting the right to a fair trial (Article 6) and to liberty (Article 5) are intrinsic to the purpose of considering fitness to plead, but there were significant concerns that the procedure was failing on both counts.

Deprivation of liberty via psychiatric detention was the most common disposal for defendants found unfit, but only around half had mental illness.^67^ The remainder were diagnosed with mental impairment and a few with communication deficits. While medical evidence was now a requirement for a finding of unfitness, the assessing doctors were not asked to consider the appropriateness of hospitalisation, and the judge had no limits on deciding whether to impose a hospital order.^71^ Yet, a hospital order for an individual with no mental disorder would be incompatible with Article 5. The inflexibility in murder cases raised particular concerns, as encapsulated by Mr Justice Richards in 2001^72^:

Those [Pritchard] criteria do not correspond directly to the criteria for a mental disorder sufficiently serious to warrant detention, and it may be possible for a person to be found unfit to be tried without his suffering from a mental disorder sufficiently serious to warrant detention. Yet once a person facing a charge of murder has been found to be unfit to be tried, there is no further consideration of his mental condition … If the jury find … he did the act charged, it is mandatory for the judge to make an admission order … The judge cannot consider whether such an order is justified on the medical evidence … This feature of the procedure does raise the question whether detention is ‘arbitrary’ in the sense explained by the ECHR.^73^

Further changes were made to fitness-to-plead laws in the Domestic Violence, Crime and Victims Act 2004.^74^ The court must now apply the provisions of the MHA, justifying detention on the grounds of mental disorder, when making a hospital order after a finding of unfitness, including in murder cases. If a patient later becomes fit to plead, the Secretary of State is now empowered to remit him for trial. Other changes include removal of guardianship as a disposal option and MHA provisions allowing the court to remand unfit defendants to hospital for reports or treatment prior to the final disposal. This raised the likelihood of a defendant being able to undergo a full trial, signalling a shift towards valuing the right to exercise legal capacity over the right to be protected from trial, which has gained momentum with the UNCRPD.^13^

## Conclusion

This journey through the history of fitness to plead illustrates that while the centuries-old principle that the mentally disordered require special legal treatment is unchanged, our understanding of how mental disorder impacts behaviour and how this can and should be managed has progressed significantly over time. The competing demands of individual rights versus public protection and natural justice versus punishment have carried different weight at different points in history. The primacy of natural justice and treating the insane with leniency gave way to the need for public protection following high-profile cases. The expansion of Victorian asylums preceded improvements in conditions and treatment for inpatients, and individual rights were poorly served until at least the first iteration of the MHA in 1959. Improvements in understanding and treatment of mental disorder combined with the ascendance of human rights legislation have tilted the balance somewhat back in favour of individual rights. However, the test for determining unfitness remains problematic. It evolved in a piecemeal fashion without due consideration of the normative abilities required at trial. The law has bestowed a leading role to psychiatry in determining and managing unfitness to plead, but modern psychiatry and 18th-century case law do not make a good fit. It is beyond the scope of this paper to critique the proposals made by the Law Commission, and until the law is changed, it is unclear how far any new test will go to reconcile the legal framework for fitness to plead with its intended purpose.
